# Perceptions of general practitioners towards the use of a new system for treating back pain: a qualitative interview study

**DOI:** 10.1186/1741-7015-9-49

**Published:** 2011-05-09

**Authors:** Tom Sanders, Nadine E Foster, Bie Nio Ong

**Affiliations:** 1 Arthritis Research UK Primary Care Centre, Primary Care Sciences, Keele University, Staffordshire, UK

## Abstract

**Background:**

Changing clinicians' behaviour is recognised as a major challenge. It is clear that behaviour change not only depends on demonstrating the proven effectiveness of clinical interventions; contextual and occupational factors, such as 'change readiness', may be central to their implementation. This paper highlights the context of behaviour change in relation to a healthcare innovation introduced within primary care, highlighting the importance of organisational and interpersonal factors that may help explain the dynamics of implementation.

**Methods:**

Qualitative interviews were conducted with general practitioners (GPs) before (n = 32) and after (n = 9) the introduction of a subgrouping for targeted treatment system. GPs were offered an electronic six-item subgrouping tool, to identify patients according to their risk of poor outcome ('high', 'low') in order to help inform their decision making about treatment approaches. Recruitment was based on a 'maximum diversification sample', to obtain a wide representation of views across all five practices. A coding scheme was developed based on the emergent findings, and the data were analysed using 'constant comparison', drawing upon insights and developing connections between themes. We adopted the normalisation process theory (NPT) to explain the uptake of the new system and to examine the relevance of coherence for the implementation of innovations in organisations.

**Results:**

GPs perceived back pain as a low clinical priority, and highlighted the importance of 'practical' and 'relational' coherence in decisions to adopt and engage with the new subgrouping for targeted treatment system. Health professionals often engage in 'sense making' about new innovations to 'road test' their applicability or relevance to daily clinical routines. Low back pain was generally perceived as an 'uninteresting' and clinically unchallenging health problem by GPs, which may partly explain their lack of engagement with the new subgrouping for targeted treatment system. The adoption of this new way of working by GPs was determined by the meaning that they ascribed to it in the context of their daily clinical routines.

**Conclusions:**

We conclude that the key obstacle to implementation of the new subgrouping for targeted treatment system for low back pain in primary care was an initial failure to achieve 'coherence' of the desired practice change with GPs. Despite this, GPs used the tool to different degrees, though this signified a general commitment to participating in the study rather than a deeper attitude change towards the new system.

## Background

Interventions aimed at changing the behaviour of clinicians have had limited success [1,2]. Only a few studies in the field of low back pain have shown changes in clinicians' behaviour [3]. The assumption that rationalistic approaches to behaviour change will self-evidently become adopted in practice is essentially flawed [4], as clinicians may prefer to manage patients in accordance with established routines. Locally agreed treatment pathways may be followed in preference to national recommendations; a number of reasons have been offered for the apparently limited uptake of innovations in healthcare settings. It has been suggested that in order to affect change, a better understanding of how clinicians define 'effectiveness' is required; if healthcare professionals perceive new ways of working to be 'effective' then they may be more likely to adopt them [5]. Others, however, stress the need to examine the impact of interpersonal relationships and 'network ties' in healthcare settings on 'adoptive' behaviour [6]. Previous research has not used robust theoretical frameworks to explain change (or the lack of change) beyond the narrow focus on individual clinicians' behaviour. This is particularly significant given the current need for systematic, theoretically informed studies of the applicability of research-based knowledge to routine clinical practice [7,8].

Normalisation process theory (NPT) provides one such framework for understanding why health care interventions are accepted and employed routinely in organisations and others rejected; in particular, examining the social conditions underpinning behaviour change in healthcare settings [9]. NPT helps to explain which factors promote and prevent the adoption of innovations (at the individual level and the level of the organisation), with emphasis on early and subsequent phases of implementation. Thus, it provides a framework to examine the activities that people engage in, that lead to new ways of working and their long-term sustainability. The 'normalisation' process is divided into three stages; implementation (introduction of new ways of working in a clinical environment); embedding (routine incorporation of the new way of working by individuals or groups); and integration (reproduction and sustainability of the behaviour). Consequently, while the processes are not linear, the success of the latter two stages depends on the first, implementation, which relies on the acceptance of a social practice as 'meaningful' (coherent). Perhaps the most common obstacle to implementation is an initial failure to demonstrate 'coherence' to the users, that is, if professionals fail to perceive the new way of working as helpful and relevant they may be unwilling to deploy it [10]. Health professionals may engage in 'sense making' about new ways of working, to 'road test' the applicability and relevance to daily routines. Coherence in this context may be defined as a process that binds working practices together, resulting in consistency and uniformity of daily work [11]. Consequently, we chose to adopt the conceptual idea of 'coherence', used to explain the initial stages of implementation, to examine how the subgrouping for targeted treatment system was perceived by general practitioners (GPs) and introduced into existing clinical practices. We were interested in explaining the obstacles to the early adoption of the new system, which fitted in with the concept of 'coherence', and because we found little evidence that the other NPT constructs (for example, cognitive participation, collective action, and reflexive monitoring) accurately reflected the behaviours and attitudes of the GPs in this study.

### 'Coherence' in clinical work

It is increasingly clear that changing clinicians' behaviour does not solely depend on the proven effectiveness of the new approach or its dissemination; contextual as well as occupational and professional factors, such as doctors' desire to retain their autonomy over clinical decision making may be critical to any attempts at introducing innovations in health care [12]. Historically, the ability to control the form and content of clinical work has been the hallmark of the medical profession, allowing doctors to exercise considerable autonomy over decision making free from external intervention [13,14]. Although the UK National Health Service (NHS) has witnessed numerous reorganisations in the last decade, new information technology, computerised patient records, and most notably clinical guidelines all examples of such change, it is not axiomatic that clinicians will in turn embrace new innovations or change behaviour in line with scientific evidence [15,16]. The opposite may hold with attempts to match healthcare interventions with established clinical practices, perhaps adapting those elements that are perceived to be particularly useful to individual patient care while rejecting those thought to be less appropriate. Or, simply the embeddedness of clinical work could render change difficult to achieve [17]. Despite a few exceptions however, rarely have rigorous evaluations of contextual and strategic processes been conducted that help explain the 'institutionalisation' of new approaches to service delivery [18] which, given the current dependence on evidence-based knowledge systems in the NHS, is perhaps surprising.

However, achieving coherence for new ways of working within organisations does not automatically guarantee acceptance by professionals [19]. Behaviour change initiatives such as financial incentives have previously been adopted in primary care (Quality Outcomes Framework), having a significant impact on GPs' clinical work. However, this study sought to examine the latent dynamics present within healthcare organisations that highlight GPs' resistance or otherwise to changes in their daily work. Emphasis on the coercive external levers of behaviour change such as financial incentives may overlook such factors. This paper, based on qualitative interviews with a sample of GPs, highlights some of the contextual factors affecting the implementation of one such innovation in general practice; including such issues as interpersonal and practice-based obstacles that have received limited research attention. In particular, we examine the process of 'sense making' by GPs in relation to a new system involving subgrouping for targeted treatment for patients with low back pain, and how the acceptance of a social practice, in our case the use of a subgrouping tool, becomes defined as meaningful (or 'coherent').

Low back pain is the most common reason for consulting a GP among middle-aged people, with approximately 6% to 9% of adults consulting for this condition each year [20]. Best practice guidelines have emphasised the need to assess and manage patients according to a biopsychosocial model [21] in which key obstacles to recovery are identified and treatment incorporates both symptom management and secondary prevention. A key challenge is the early identification of patients at risk of chronicity and its subsequent prevention [22]. Brief prognostic assessment tools have been developed and validated for use in primary care to identify subgroups of patients at risk of persistent low back pain who may benefit from more targeted interventions. Although the approach has been tested in a clinical trial [23], the perceptions of GPs towards the use of the subgrouping for targeted treatment system are unknown.

## Methods

### Context

This study falls within the tradition of organisational change and uptake of innovation studies which adopt qualitative designs that are able to explore complexity, dynamics and multiple perspectives. The interviews were nested within a larger study (the IMPaCT Back ('IMplementation study to improve PAtient Care through Targeted treatment for Back pain') study), which aimed to evaluate the improvement in quality of care for back pain patients following implementation of a subgrouping for targeted treatment system. The GP interviews were embedded within a prospective, population-based, quality improvement study comprising three phases: (a) assessment of GPs' and physiotherapists' attitudes and behaviours regarding low back pain, (b) a quality improvement intervention comprising educational courses, regular feedback sessions, and the installation of computerised and paper-based systems for subgrouping for targeted treatment system, and (c) assessment of GPs' and physiotherapists' attitudes and behaviours regarding low back pain and the subgrouping for targeted treatment system [23]. Currently there is no systematic screening of back pain patients in primary care to help inform who should be managed by the GP and who should be referred on to other health professionals such as physiotherapists. A subgrouping for targeted treatment tool specifically for primary care has been developed and validated [24]. Two GP leads were fully engaged in the early design and piloting of the subgrouping for targeted treatment system, and played a major role in discussions with the research team and dissemination activities with clinical colleagues at the five GP practices. The GPs helped design the six-item subgrouping tool through involvement in working group meetings during the development stage, which was pilot tested in one of the five GP practices.

The IMPaCT Back study was located in five GP practices (and associated physiotherapy services) within one Primary Care Trust. The practices range in terms of size (populations of 4,000 to 24,000) and composition (4 to 12 GPs and a variety of primary care services). Taken together they are representative of similar English general practices. The five practices were identified from one Primary Care Trust, which is part of a larger GP network with which the research centre has collaborative links. The practices were selected on the basis of varying practice sizes (medium and large, but not small), geographical locations (rural and town, but not urban), and willingness to work with the research team on studies investigating musculoskeletal problems. The five practices also represented deprived, affluent, and mixed (both affluent and deprived) patient populations. The practices use various physiotherapy pathways, but, again, represent the national picture.

The literature shows that primary care management of low back pain is inconsistent and outcomes are often poor. Similar patients with back pain in primary care can receive different treatment approaches by GPs, some will be referred on to other clinicians such as physiotherapists, and others not. The new subgrouping for targeted treatment system is intended to assist GPs in the identification of patients at risk of persistent problems, who may benefit from targeted interventions. We envisaged that GPs would find such an approach helpful because it would lead to more systematic identification of patients at high risk of poor outcome, for whom early access to physiotherapy may be more helpful. Conversely, the tool could assist GPs in feeling confident about what they had to offer those patients identified as at low risk of poor outcome, without the need for onward referral. To add to the flexibility of clinical management, GPs were given the option to follow the advice of the tool which included both referral to physiotherapy or best GP practice for back pain; and the option to record their own clinical impression of the patients' risk of poor outcome. GPs and physiotherapists were each given additional training in the new subgrouping for targeted treatment system and the electronic subgrouping tool was tested in one GP practice prior to installing it in the others. The tool had a direct relevance to GPs' daily management of back pain, as it offered assistance with identifying patients for whom onward referral to physiotherapy might be most appropriate. All participating GP practices and GPs were offered the subgrouping tool following an observational phase in which current practice and patient outcomes were monitored.

### The intervention

In phase 2 of the study GPs were offered a six-item subgrouping tool, to subgroup patients according to their risk of poor outcome ('high', 'low') in order to help inform their decision making about treatment approaches. Patients at low risk of poor outcome were recommended to receive best primary care advice and management by the GP including red flag screening, reassurance and advice about pain relief, activities and work, supplemented with a brief information sheet, based on recent guidelines [25]. Those at high risk of poor outcome were recommended for referral through to physiotherapists who could identify and manage patients' physical and psychological obstacles to recovery. The decision tool was provided in two forms, an electronic version embedded in their clinical computer system that was activated when a back pain Read Code was entered, and a paper-based version for those GPs who wished to use it.

### Interviews and recruitment

Here, we only report the interview findings with GPs. We conducted 32 qualitative, semistructured interviews (some by telephone) with GPs at baseline (prior to the introduction of the subgrouping for targeted treatment system) from a total of 65 GPs working at the 5 practices (see sampling procedure below). The telephone interviews were on average shorter than the face-to-face interviews, but covered the same issues. All GPs were invited to participate in the first stage interviews, 32 of whom agreed to an interview. Consequently, 33 GPs either did not reply or declined to take part. These interviews aimed to elicit in-depth information about GPs' experiences of managing patients with low back pain, to help identify the clinical challenges and potential obstacles to change in clinical practice, though they were relatively brief (approximately 10 to 15 min long). In addition, 24 GPs were invited to participate in a further in-depth interview 12 months after the subgrouping for targeted treatment system had been introduced in the practice, and 9 agreed. These second interviews were designed to explore in more detail their experience of using the subgrouping tool and its recommendations about onward referral, its perceived value and fit with their clinical practice and the reasons for use and non-use. We did not contact all of the original 32 interviewees, as several had indicated during the first interview that they did not want to participate in further interviews. A total of 9 GPs finally participated in the second interviews, while the 15 GPs who declined did so without giving a reason. GPs who agreed to a second interview were, on average, higher users of the subgrouping tool (interview participants used the tool with, on average, 47% of their low back pain patients compared to 30% in those who did not participate in the second interviews).

The GPs were selected in two phases: prior to the implementation phase of the subgrouping for targeted treatment system in the study, and after the implementation phase was completed. Recruitment was based on a 'maximum diversification sample', to obtain a wide representation of views across all practices. In the first phase in-depth data on GPs' routines as part of managing patients with back pain was obtained, while the interviews in the second phase focused on decisions to use the subgrouping tool and its recommendations about onward referral in practice, and what impact, if any, the tool had on GPs perceptions and behaviour. Semistructured interviews were organised around the four dimensions of the NPT: 'sense making' about new interventions by professionals, the division of labour in primary care, dissemination and interpretation of knowledge, and the organisational and social context. These four elements provided a framework with which to examine the conditions that make 'normalising work' possible, as well as the factors that impede this process.

### Analysis

All interviews were tape recorded and fully transcribed. Analysis was aided by the NVivo data management system (http://www.qsrinternational.com/products_nvivo.aspx). The constant comparative method [26] was the primary analytical tool. The interview transcripts were coded independently by three researchers to agree a coding frame. A coding scheme was developed based on the emergent findings, and the data were analysed drawing upon insights and developing connections between themes. NPT was adopted as the guiding theoretical framework underpinning emerging themes and concepts. However, NPT was used primarily to guide analysis, and not to restrict the exploration of other possible theoretical insights, such as through a broader application of the research and theoretical literature to the data. The emergent insights from the interviews were summarized in descriptive form, and used to test out potential hypotheses during subsequent interviews. This process assisted theoretical development, and continued following the completion of the data collection phase. The analysis of the second stage interviews identified seven emergent themes, which were mapped onto the 'coherence' construct within the NPT. The themes are summarised as follows: (a) the tool as a low priority, (b) the tool did not account for complexity of clinical decision making, (c) GPs lacked familiarity with the tool, (d) lack of incentives to use the tool, (e) limited perceived impact on clinical decision making, (f) limited peer discussion about the tool, and (g) perceived high impact of tool on peer communication. We interpreted these findings as representing a lack of 'coherence' to GPs of the new system. The emergent themes did not map well onto the other three constructs of the NPT. This is because the subsequent three dimensions of the NPT (cognitive participation, collective action, and reflexive monitoring) presuppose a high degree of 'coherence'. Team meetings were held regularly to discuss emergent findings and to ensure their consistency and reliability. Full NHS ethics approval was granted by Cheshire Research Ethics Committee (reference: 08/H1017/65). Research governance clearance was also obtained.

## Results

### GPs' constructions of 'routine' back pain work (interview findings preintervention)

There were no obvious differences in the views expressed by GPs who followed the recommendation of the subgrouping tool and those who did not. In the majority of cases GPs demonstrated a relatively high level of non-adherence to tool use. This finding is strongly borne out in the interviews with GPs who had not used the tool expressing similar views to those who had used the tool, citing the main themes (reported below) as the main reason for their behaviour.

The main focus of these first interviews was on GPs' current approach to back pain, and the issue of routinisation was central to their accounts. The GPs described non-specific low back pain (LBP) as a 'common' complaint that, when compared to the major chronic illnesses such as heart disease or diabetes, had a lower priority for them. GPs followed their own 'script' for all patients with LBP symptoms, allowing them to classify their complaint according to pain severity and follow a management plan which initially included information and exercise advice, followed by subsequent referral to a physiotherapist for those with persistent symptoms. Such a strategy could detract from exploring patients' unique account of their condition and experiences. Most GPs were confident in their ability to match their patients' symptoms with what they perceived to be the appropriate treatment pathway. By and large decision making seemed to be heavily 'scripted'.

GP13: 'Erm, generally I feel OK, in the sense that I am pretty clear about what I want to do with most of the patients. The vast majority, have probably got a self-limiting back problem, so chronic pains, and I have a good idea in terms of medication that I want to intervene with...'

GP14: 'I am confident that I know the right investigations to do and who to send them to for further advice, physios or orthopaedic and I am happy that I can pick out if there is a serious problem, but I am not specialised or I don't have any special interest in the back.'

Most GPs claimed that many patients with low back pain do not require a consultation with a GP, given that the problem is often reasonably self-limiting.

GP24: 'From my point of view I do feel I see a lot of people unnecessarily with simple lower back pain that doesn't necessarily require a GP appointment.'

GPs were reluctant to spend much time managing low back pain, reflected in the limited time they typically spent with patients during consultations, preferring to 'dispose' of patients [27]. One strategy was to refer them to physiotherapy, occupational therapy or to other clinicians.

GP15: 'I think one of the difficulties I find is, if someone has a chronic back pain, I wish there were occupational physicians that we could say 'oh maybe you could go and see someone if it is affecting your work'.'

GPs also reassured patients that their symptoms were likely to be muscular and therefore self-limiting; a strategy used perhaps to minimise repeat consultations.

GP18: 'When they are in acute pain, when it's just happened, I think a lot of people are thinking, 'oh, I have broken my back', 'I've slipped a disc', or whatever and to be reassured at that point that it is more likely to be muscular and will settle, they are just glad to be reassured, but I always put in the proviso, but if it's not improving, or you get any red flags, to come back straight away.'

Others expressed conflict between their own and patients' expectations of treatment.

GP24: 'I think people are much, they much more want a sort of quick fix and they want a cure, rather than being prepared to take some responsibility, and follow the doctor's advice, and take exercise, lose weight, that sort of thing. So it's a bit frustrating because often you'll see people and they'll say 'Oh I've had back pain' and you'll see that they've DNA-ed (did not attend) the physio you know sometimes, or whatever, so probably for back pain I think that patients are less than committed often, and that's frustrating.'

GP30: '...people who have got chronic back pain want to have a diagnosis, and that's impossible to give most of them and they find it very difficult to accept that what you're trying to do is control and improve symptoms rather than cure them.'

GPs viewed patients' lack of understanding about the typical trajectory of back pain as a hindrance to patient recovery rather than an opportunity to learn about their experiences and how they relate to their perception of the problem and its secondary prevention.

GP7: 'I think the patients often perhaps don't appreciate the natural history [of back pain]... when we are just waiting for the natural history to evolve, isn't accepted very well, very favourably by the patients.'

Wider organisational and professional issues had a significant impact on back pain management, with GPs often reluctant to refer patients to physiotherapy services.

GP11: 'I am sure that physiotherapists are the most appropriate practitioners to treat back pain, so the only thing that stops me is use of resources... yes almost everybody with back pain will benefit from that, but we would run out of physiotherapy resources very quickly when we need it for other things.'

Likewise, the need for orthopaedic services to manage only the most complex low back pain cases resulted in a widespread reluctance by GPs to refer patients; referral in such circumstances could risk damaging professional credibility or even relations with colleagues in secondary care.

GP13: 'the orthopaedic people, who are just quite laid back about everything unless it is a barn door sort of [thing] standing up in front of you, they are just not really interested. So sometimes I think where I get a bit stuck with back pain is when the secondary care services and the registrars, or, they have got quite a high threshold for taking admissions... when you have the acute backs... in an ideal world, with some of those people you want an instant MRI scan to see what is going on and that's just not possible.'

After the first (observational) phase the GPs were introduced to the subgrouping for targeted treatment system and the six-item subgrouping tool through practice-based meetings with clinical opinion leaders and the study research team. Prior to offering the tool to all practices, it was piloted in one practice and found to be simple to use and acceptable, adding no more than 2 min to the consultation. The use of the tool was supported by follow up visits to the practices by informatics staff, to ensure the computer 'pop-up' screens were working and to provide regular feedback (every 2 months) via email and letter about number of patients with low back pain seen and for whom the tool had been used. Offers of additional guidance were made to practice managers and link GPs at each of the participating practices, in these feedback communications. The research team clearly discussed how they would monitor referral rates to physiotherapy and provide additional resource if and where needed. The tool provided the GPs with more systematic information about their patient's prognosis and gave a treatment recommendation based on each patient's subgroup classification, in the hope that patients at risk of poor outcome would receive earlier targeted treatment to help improve their outcome. Subsequent interviews were conducted approximately 12 months after the introduction of the new subgrouping for targeted treatment system in each GP practice.

### Practical coherence (interview findings postintervention)

We use the term 'practical coherence' to denote the extent to which clinical routines and behaviours affected GPs' ability to use the subgrouping for targeted treatment system. By 'practical' we refer to those behaviours that enable a task to be managed efficiently, within the constraints of a busy clinic. The GPs perceived the subgrouping tool to have a lower priority relative to other tasks (for example, Quality and Outcomes Framework (QoF), national and local targets), and consequently claimed that time constraints and pressures intrinsic to a busy practice prohibited them from using the tool. Thus, the extent to which the tool could enhance existing clinical routines appeared to strongly influence tool use.

GP35: '...the problem is the time. It's definitely it's playing a major [part] on the issues you know. You're doing something a bit extra on top of what you normally do on a daily basis, which probably already a bit struggling with so timing probably is the most important thing you know.'

GP17: '...it's (the tool) not had very much visibility in the last year (laughing). When I'm talking about a 2 and a half million overspend, I'm afraid the IMPaCT Back study didn't have much of an impact. It might have a million pound public health resource but you know, but at my level it was I'm looking at overspend.'

The following respondent claimed that despite the simplicity of the decision tool, it was rarely used because the task of examining patients and discussing their problems took priority in the context of a busy working schedule.

GP35: 'You've got to examine, you've got to document everything you know, whether they've got any sort of red flags. These all take time... when you've got just about 10 minutes even the seconds is really important and probably that was something that I struggled [with] and if I couldn't really discuss anything in that particular time you know, it was just a timing issue but nothing else...'

A major finding from the interviews was that GPs felt the tool did not account for the complexity of decision making in low back pain. The following GP stressed the impact of patient demand on referral decisions, which is not taken into account by the subgrouping tool.

GP3: 'Whereas, with a patient it's more of, I don't know how to put it really, it's not like a bartering but it is a bit like that you know. They want physio, well sometimes they want physio, and you're thinking well you know, should they have it or not and they may only score one but if they really want physio, they going to get physio because we're here to meet the patient expectations and demand...'

The following GP claimed that medicine was an art, requiring awareness of the human elements of medical care, which the tool could not effectively capture.

GP3: 'It's difficult to say because you see, I'm one of these that thinks that there's an art to general practice and it's more a sort of conversation and a feeling between two people. Now, you can't put feelings into a questionnaire [reference to the tool] so what's right for one, is completely wrong for another and unless you had a questionnaire that was a thousand questions long, you're just not going to capture that, are you? ... It's not a tool that I would want to use within my consultation because it's not how I practice but it's not been obstructive to me.'

Others found that the tool conflicted with established patterns of care, encouraging what they saw as 'inappropriate' referrals to a physiotherapy service that was already struggling with waiting times.

GP10: 'The times I've used it, I've brought it out and said all right to the patient, '[X University] are doing a study let's look at this' you see. So you go through it and then it says 'referral to physio', and so that puts the idea into the patient's mind which you then can't go back on, and I have to say that the very few that I've done, were not people I would have immediately referred to physio, and our physio team can't cope at the best of times you know. They're about a month behind...'

Other GPs found it difficult to alter their routines to fit in with the decision tool, and often forgot or refused to use either the computer or paper-based tool during clinics. Even those GPs that asked for the paper-based tool appeared to struggle to remember to use it with patients.

GP11: 'My major difficulty was remembering to ask the questions because my practice is to enter my notes after the patient has left, so often I would then, you know three or four problems you put them all in, you give the back pain one and the (computer) template throws up and you think oh I've forgot to ask them.'

GPs expressed a general lack of familiarity with the subgrouping tool, claiming that they would have benefited from more frequent contact with the research team for feedback and guidance.

GP17: 'Certainly. I haven't accessed your tool for about a year to be honest, so my familiarity with it, which is part of the reason why last time we spoke, I spoke to someone, I said I would engage more but to be fair, it's probably a lack of familiarity at that point that just hindered the process.'

GP3: 'So that's not been an issue. It's not a time factor; it's remembering to do it if the computer's not prompting you.'

However, involvement in the research led some GPs to reflect on their current practice, which in itself appeared to have a positive effect on behaviour.

GP11: 'There were occasions when it made me rethink and I thought oh well, perhaps I should, because sometimes the process; none of us are consistent and sometimes you don't do the best job so, sometimes you've made a fairly quick decision and, you think you've got to move onto other things, other things are more urgent; more pressing so you haven't necessarily done the best job and the discipline of asking somebody those questions makes you rethink and you think actually perhaps I haven't done as good a job there as I would.'

Several GPs wanted more information about the physiotherapists' new role in the subgrouping for targeted treatment system.

GP13: 'But I wasn't I think initially when [X] may have presented, you know even before recruitment, I think they talked then about what they were looking at but I think it would have been useful as part of Y's and Z's [research team] presentation, to maybe have a physio there saying that this is you know, what we will offer as the intervention.'

GP13: 'Hum, yes in a roundabout way. So I know about the assessments that they [physiotherapists] do and also the psychological therapies that they're sort of putting in but not, I couldn't, if you said to me could you write an essay or a side of A4 of all the things that they do, no I couldn't...'

### Relational coherence

Relational coherence refers to the impact of the subgrouping tool on interpersonal relationships between peers. For instance, the use of a new system for targeting and treating patients may affect referral decisions to certain services and directly impact on their workload, or the extent to which tool use is reinforced within an organisation through informal discussion, debate, and (dis)agreements between peers. Such activity may be a prerequisite for the introduction, wider acceptance and 'legitimation' of any new innovation in health care. A central issue affecting the acceptability of the subgrouping tool was the presence or absence of peer communication. Communication between colleagues served two aims: (1) raising awareness (often more important in larger organisations where dialogue between individuals may be less direct), and (2) individual or collective evaluation of a new way of working (integral to its acceptability). The GPs claimed that they generally did not discuss the decision tool or subgrouping for targeted treatment approach with colleagues.

GP11: 'The only time that we've really discussed that has been when you've been to see us.'

Interviewer: '...you ever got chance to have a real good chat with your colleagues as far as what you've been asked to do and how you felt about it?'

GP3: 'I mean we have so many things to discuss, but no, minimal I would say.'

GP10: 'I mean we perhaps talk for about 2 or 3 minutes after you've been to see us (laughing) but then it gets forgotten until the next time if I'm honest.'

Another GP was unclear about his role in the study, claiming that he would like to have received a formal 'contract' with the research team about his role and obligations, which would have improved communication with colleagues.

GP13: 'This is what you're signing up for. Erm, I would have been happy to do it. It wouldn't have been a problem but I would have been much clearer about my role and then I was getting newsletters or feedback from yourselves. I would have understood it was my role to disseminate the information and to discuss it at the, you know, the PLT [Practice learning time] or the lunchtime briefs that we have, and also to share the recruitment figures and to feedback to my partners where we were up to. I think that would have cascaded then.'

GPs discussed the tool together when concerns were raised about the impact that it had on their practice or on the referral rate to physiotherapy. The following GP claimed that discussion with peers resulted in a practice wide consensus that the tool was inappropriate as it recommended that too many patients be referred to physiotherapy.

GP9: 'Certainly in the earlier part of the study, I was referring anybody who came out with a score of 3 or greater but having sort of reviewed that as a peer group of doctors here and discussed whether we should be doing that, we actually came to the conclusion that there are a lot of unnecessary referrals being done purely because of the score 3 or more; ordinarily wouldn't need physiotherapy and probably would benefit relatively little from having it.'

GPs claimed that referral to physiotherapists was conditional on receiving regular feedback on patient progress and timely discharge.

GP13: 'I don't make assumptions about patients but I would [I think] feel more confident about referring patients who I think are going to be left with long-term sick really with an acute presentation so I have a lower threshold for referring patients to physiotherapy, as long as I knew that we were going to get the additional input. I think I'd be reluctant to refer patients with a low threshold who I felt were then going to be attached to physiotherapy, you know, for a long time.'

The danger of overwhelming physiotherapy services with patients presenting with underlying psychological health problems was mentioned by some GPs who felt that any decision instrument that encouraged referral of this nature raised concerns, and was less likely to receive support from GPs.

Interviewer: 'So what sort of clinician do you feel is the most appropriate to deal with patients that consult with low back pain that have the psychosocial issues?'

GP9: 'Traditionally it's been GPs because things like that tend to fall on GPs as sort of the gatekeeper to other services. I think there's no problem I would have with them seeing a physiotherapist as a first port of call but I think the physios would have overwhelming concerns about that because they really don't have the capacity to do that on the NHS.'

A similar view was raised by the following GPs, whose referral decisions were affected not only by the patient's physical complaint but also by the potential burden on physiotherapy services and consequent relations with physiotherapists [10]. They obviously did not appear to have fully understood that additional physiotherapy resources were made available as part of the study, which perhaps raises questions about the communication process between the study team and the participating GPs. It is clear though that engagement in the new system required more than financial resources.

GP35: 'There was a constant struggle you know with the physio team because they were under a lot of pressure and you know, maybe as a GP you know, you were a bit under pressure even morally you know should I? Shouldn't I? You know, is it better or not, you know? What's the best basically to do? You know, this kind of struggle that you normally have and in sort of real life, you know sometimes the things that look really really nice on paper you know and in real life you know, sometimes it doesn't work unfortunately.'

GP10: '...I wasn't going to get my wrists slapped, because you do sometimes get your wrists slapped. There's one physio in particular that just bounces them. So you have to justify every referral really...we get messages saying what the wait is for physio and you know, please don't overload us and we're often being told that so I try and manage. I have to say, I try and manage the psychosocial side myself if I'm honest.'

## Discussion

The task of integrating new ways of working in healthcare settings can be challenging [28]. According to the NPT the implementation of a new approach is operationalised through four mechanisms: coherence (establishing meaning), cognitive participation (engagement of individuals); collective action (interaction with existing practices); and, reflexive monitoring (reflection and understanding of an intervention). The GPs in this study did not progress beyond the first stage of implementation, or coherence, the main focus of this paper. Coherence, or 'meaning', in practice may be difficult to achieve for several reasons. The work of healthcare professionals is often 'routinised' making it difficult to deviate from existing practices. In this study, low back pain was perceived by GPs as a common complaint, often considered to be a clinically 'uninteresting' condition, and deserving of less attention from clinicians in contrast to other health problems, which are perhaps more clinically challenging. GPs utilised well rehearsed management strategies for back pain which typically included the routine 'classification' of patients' symptoms into 'acute' or 'chronic' categories, leading to highly structured approaches; the first included predominantly advice about exercise and lifestyle, while the second involved referral to a physiotherapist for more intensive interventions. Neither typically led to a detailed exploration of the patients' experiences of their illness episode as means of guiding the decision-making task, even though in a different back pain study GPs claimed to personalise care [29]. The implications are significant. For any strategy aimed at implementing a new way of working in health care, the importance of understanding clinical routines is clear. Although individual perspectives about the care of patients varied, it was evident that our GPs followed a 'script' that contributed to the 'standardised' approach taken. They appeared to follow the recommendations set out in the UK National Institute for Health and Clinical Excellence (NICE) Guideline for Low Back Pain (2009) in their daily interactions with patients, reinforcing the importance of activity, but providing only limited guidance about the long-term consequences of the condition [21]. While asserting the need to address the psychosocial impact of back pain, the guidance offers limited practical recommendations. The consequence of this uncertainty may be important, perhaps reinforcing GPs' perception of back pain as a problem for which little can be done and for which patients should perhaps assume greater personal responsibility. GPs felt that their current approach to back pain appeared to work, as management often led to the effective 'disposal' of the patient [27].

Despite a successful pilot at one practice, the GPs claimed that the subgrouping tool did not integrate well into their everyday management of patients. The organisational pressures affecting the care of patients, such as time constraints presented major obstacles to the use of the decision tool. The perceived motivation of patients to adhere to clinical advice has been shown to affect GPs' decisions to incorporate scientific evidence in practice, as reported previously in relation to smoking cessation [5]; if patients fail to follow advice then GPs might be less likely to offer it routinely. In this study GPs claimed that clinical decisions frequently involved making subjective judgements about patients' likely motivation to act upon advice, where medical decision making was an 'art' often influenced by subjective considerations. In this regard the translation of evidence into practice is rarely straightforward, since it demands an awareness of patient-specific influences affecting outcomes. Other obstacles included GPs' apparent desire to avoid overloading physiotherapy services (even in the context of the service actively participating in the IMPaCT Back study and accessing additional support to do so), while adherence to the decision tool was perceived to have the opposite effect; increased referrals. Coherence in this context might therefore be defined in terms of 'role congruence' between existing healthcare routines and resource concerns and the extent to which they are 'disrupted' by an innovation [30]. Clearly, a distinction exists between health professionals' willingness to participate in a research study and the adoption of new tools in routine clinical practice. Several GPs were unclear from the start if they were only required to recruit patients into the study via the tool or to also use the tool to improve their daily clinical practice. This raises important questions about recruitment of health professionals into implementation research, and the strategies required to maximise engagement; for instance, through discussion about the goals, benefits and potential limits of the study.

Peer communication also seemed to affect GPs' 'adoptive' behaviour. There is clearly some overlap between the themes reported here, though it is important to point out that there were different dimensions to the pressures prohibiting GPs from routinely using the tool. GPs perceptions about the economic consequences of their referrals to physiotherapy services were a factor affecting whether or not to adopt the tool. Occupational factors, including concerns about the displacement of labour to other groups of workers (in this case, physiotherapists) were another. The risk of undermining professional relationships with peers, for instance, as a consequence of over referral to physiotherapy, was a concern for some GPs. The same GPs incidentally challenged the suggestion that physiotherapists were the most appropriate professionals to manage patients with back pain, particularly those with more complex 'psychosocial' problems; claiming that in such cases patients would ideally require the attentions of a GP, though they expressed this view through a 'managerialist' discourse apparently concerned with the protection of physiotherapy services. This finding might be viewed differently, perhaps as an attempt to protect occupational work boundaries; GPs' reluctance to surrender control over patients could be a strategy aimed at defending their role and location in the status hierarchy. Thus, resistance to using the decision tool in practice may be a symptom of an underlying occupational desire to protect health care jurisdictions [31]. A small number of GPs understood the enhanced role of the physiotherapists in the subgrouping for targeted treatment system and saw this as an opportunity to shift the care of back pain patients who exhibited significant obstacles to recovery to specially trained physiotherapists. The subgrouping for targeted treatment approach aimed to help GPs decide which patients were most likely to need onward referral. They were not always concerned with maintaining boundaries, but considered alleviation of their burden of work, especially with regard to potentially 'intractable' patients as a positive step.

Clinicians have historically been sceptical about the utilisation of formal, rationalistic measures to support the delivery of care, particularly if they perceived a threat to their clinical freedom. Pope [32] claims, in relation to the routine adoption of evidence-based guidelines that 'By privileging technical knowledge that can be formulated and specified...EBM (evidence-based medicine) thus presents a significant threat to clinical judgement and ultimately control over medical work' [32]. Resistance to healthcare interventions could be taken as a reflection of clinicians' desire to maintain control over their work [30], with the intervention (which might include extended roles for other groups, such as physiotherapists) perceived as a direct threat to that autonomy. Equally, guidelines may enhance autonomy by presenting clinical decisions as scientific [33]. Our GPs, however, rejected the standardised rules embodied by the subgrouping tool, questioning its ability to significantly empower them or enhance their practice in the management of back pain. Coherence in this regard could be viewed as the degree to which a healthcare intervention is perceived to be a threat to an occupation's influence over a sphere of work. Various mechanisms of professional closure may be deployed to aid this task and to safeguard control over services or labour.

Medicine's claim to autonomy, to set its own standards and control clinical performance without outside interference, is its central characteristic [34]. Of course this principle can be compromised as primary care commissioners (such as Primary Care Trusts in England) are often able to exert a major influence over the context of clinical practice, such as through control over resources. Similarly, patient demand can affect clinical decision making and shift the balance of power away from doctors. Yet, for all these constraints, the cornerstone of the medical profession is its claim to clinical autonomy, and emphasising its importance lies at the heart of professional work despite the threats. Clinicians defend what may seem like irrational behaviour, in order to exercise 'discretionary power to cope with various patient, practice and workload pressures' [35]. This position, far removed from the usual behavioural levers of coercion and economic incentive renders any change to their behaviour difficult to achieve [35]. Consequently, the claim that discretionary power was necessary to manage the pressures of everyday clinical practice was implicitly made by our GPs in relation to the subgrouping tool. If the tool failed in its ability to at least assist the task of managing patients then GPs could refuse to use it, irrespective of its effectiveness. Our study shows that even when an innovation is developed by, and in collaboration with, those for whom it is intended, this does not guarantee its integration in practice [19]. In the final analysis, it is difficult to conclude whether physician resistance, the subgrouping tool itself, or organisational factors had the greatest effect on the adoptive behaviour of clinicians. It would appear that a combination of all three had an important impact. However, underpinning these are the practical and interpersonal dimensions of routine general practice that may be the strongest predictors of behaviour change. In particular, the relevance and benefit of the new system ('coherence') to existing clinical work seemed to weigh heavily in the uptake of the tool.

## Conclusions

In summary, low back pain was generally perceived as an 'uninteresting' and clinically unchallenging health problem by GPs, which may partly explain their lack of engagement with it. The adoption of a new way of working by GPs was partly determined by the meaning that they ascribed to it, and any perceived change to the stability and continuity of routine medical work could be met with resistance. Therefore, an appreciation of such routines is the first step towards understanding the perceived acceptance of innovations. The second is a familiarity with how a new way of working may affect work patterns; and, the third is the impact that it may have on interpersonal relationships with peers. Failure to adequately understand all three dimensions may result in largely unsuccessful attempts at integrating new ways of working in the NHS.

## Limitations

Recruitment of GPs into health research is notoriously difficult [36]. The nine interviews that were conducted lasted between 30 and 40 min on average and taken together provide a large amount of data. The final sample size is a reflection of the recruitment difficulties that were encountered, though the analysis provides theoretically informed, critical insights into the reasons preventing GPs from using the new system, with important implications for future studies. Moreover, their value comes from the insights rather than from the quantity of data collected. The initial interviews that we conducted by telephone inevitably involved a compromise, as we wanted to obtain a wide range of views from a large number of GPs about their daily routines. At this stage our aim was to obtain insights about the variety of issues facing GPs regarding their management of patients with low back pain, and such breadth of opinion could only be obtained through telephone interviews. Face-to-face interviews would have taken longer with a much smaller sample of respondents.

## Competing interests

The authors declare that they have no competing interests.

## Authors' contributions

TS led the analysis and drafted the manuscript. NEF and BNO made a substantial contribution to the conception, design and analysis of the study, and were involved in manuscript development and revisions. All authors read, contributed to, and approved the final manuscript.

## Pre-publication history

The pre-publication history for this paper can be accessed here:

http://www.biomedcentral.com/1741-7015/9/49/prepub
